# Maintaining ambulatory care capacity through telemedicine during wartime: insights from a tertiary center’s experience

**DOI:** 10.1186/s13584-026-00783-y

**Published:** 2026-09-09

**Authors:** Shachar Shapira, Danny Epstein, Meital Zur, Gilad Twig, Gad Segal, Yitshak Kreiss, Galia Barkai, Yael Frenkel Nir

**Affiliations:** 1 Management Wing, Sheba Medical Center, Ramat Gan, Israel; 2 Department of Emergency & Disaster Management, School of Public Health, Gray Faculty of Medical and Health Sciences, Tel Aviv University, Tel Aviv, Israel; 3 Israel Defense Forces Medical Corps, Tel Hashomer, Israel; 4 Israel National Center for Trauma and Emergency Medicine Research, Gertner Institute for Epidemiology and Health Policy Research, Sheba Medical Center, Ramat Gan, Israel; 5 Critical Care Division, Rambam Health Care Campus, Haifa, Israel; 6 Ruth and Bruce Rappaport Faculty of Medicine, Technion, Haifa, Israel; 7 Institute for Drug Research, School of Pharmacy, Faculty of Medicine, The Hebrew University of Jerusalem, Jerusalem, Israel; 8 The Institute of Endocrinology Diabetes and Metabolism, Sheba Medical Center, Ramat Gan, Israel; 9 Department of Preventive Medicine and Epidemiology, School of Public Health, Faculty of Medical and Health Sciences, Tel Aviv University, Tel Aviv, Israel; 10 Incumbent of the Hella Gertner Chair for Research in Hypertension, Faculty of Medical and Health Sciences, Tel Aviv University, Tel Aviv, Israel; 11 Gray Faculty of Medical and Health Sciences, Tel Aviv University, Tel Aviv, Israel; 12 Education Authority, Sheba Medical Center, Ramat Gan, Israel; 13 Department of Internal Medicine, Sheba Medical Center, Ramat Gan, Israel; 14 Sheba-Beyond Virtual Hospital, Sheba Medical Center, Ramat Gan, Israel; 15 Dina Recanati School of Medicine, Reichman University, Herzliya, Israel

**Keywords:** War, Ambulatory services, Telemedicine, Hospital capacity

## Abstract

**Background:**

A state of war has the potential to profoundly disrupt routine, essential ambulatory hospital services, negatively impacting both acute hospitalizations and continuity of care. During June 2025, a war ignited between Israel and Iran, causing multiple casualties and substantial damage to essential healthcare facilities. This study examined the impact of the war on ambulatory visit volumes at Israel’s largest tertiary medical center and assessed the contribution of telemedicine encounters to mitigating conflict-related disruptions.

**Methods:**

This is a retrospective analysis of ambulatory service data collected at Sheba Tel HaShomer Medical Center between June 13 and June 24, 2025, compared to the corresponding period in the previous years (2021–2024), with a focus on the methodologies and managerial decisions implemented. We conducted an interrupted time series analysis using annual outpatient data from matched dates in 2021–2025. We modeled the proportion of visits delivered via telemedicine using segmented linear regression, incorporating calendar year as a continuous time variable and an indicator marking the onset of the 2025 regional war to estimate the immediate change in telemedicine use, adjusted for pre-war trends.

**Results:**

Between June 13 and 24, 2025, Sheba Medical Center conducted 53,158 ambulatory visits, of which 9,478 (17.8%) were via telemedicine. Half of the patients were male, and 47.8% were aged 25–64 years. From 2021 to 2024, telemedicine use increased gradually from 2.5% to 5.0% of visits. In 2025, utilization rose sharply following the Israel–Iran conflict. Interrupted time series analysis estimated a pre-war annual increase of 0.86 percentage points, with a 12.2 percentage point level change during the war (95% CI 9.0–15.5). Compared with 2024, total visits declined 20.0%, in-person visits fell 30.8%, and telemedicine visits increased 182.9%. The largest telemedicine growth occurred in cardiovascular (463.6%), pediatric (434.4%), and neurology/neurosurgery (350.5%) divisions, while pediatrics and psychiatry had the highest proportions of remote visits (40.2% and 33.9%, respectively).

**Conclusions:**

A robust telemedicine infrastructure and well-established operational methodologies developed during peacetime may enable the continuity of ambulatory services during wartime. The rapid expansion of telemedicine capacity helped minimize the overall reduction in healthcare services available to the community.

## Background

War, amongst other disasters, profoundly disrupt ambulatory hospital services by reducing routine outpatient visits, delaying or canceling elective procedures, and redirecting hospital resources toward acute and emergency care. Such catastrophes might also damage infrastructure, interrupt supply chains, decrease the number of available therapists and strain healthcare systems, often necessitating the reallocation of personnel and resources away from outpatient services. Such disruptions frequently result in the closure or reduced operative levels of ambulatory care facilities, exacerbating pre-disaster health disparities, particularly among vulnerable populations [1]. Civilian hospitals located within or in the vicinity of conflict zones have reported marked declines in elective surgeries, emergency admissions, outpatient visits, and overall revenues, as their wartime efforts concentrate on managing trauma and acute-care cases [2]. Despite these challenges, sustaining and restoring ambulatory services during and after disasters is essential for preventing avoidable hospitalizations and maintaining continuity of care [3]. Notably, prior familiarity with competencies and experience with platforms of telemedicine has been linked to reduced post-disaster emergency and inpatient utilization for ambulatory care - sensitive conditions, highlighting the potential of robust telehealth infrastructure to buffer the adverse effects on outpatient care [4].

On June 13, 2025, Israel launched a preemptive, comprehensive air operation targeting Iranian nuclear facilities and selected military infrastructure. In retaliation, Iran carried out missile strikes on both military and civilian targets across Israel, including the Israeli metropolitans of Tel Aviv, Be’er Sheva, and Haifa [5]. These events resulted in a reduction in ambulatory services, both within community–based settings and in medical centers across the state. Notably, Soroka Medical Center in Be’er Sheva was struck on June 19, causing extensive damage and numerous injuries [6]. At least 28 people were killed and more than 3,000 injured in the attacks, which also damaged hundreds of residential buildings and critical civilian infrastructure [7].

In the current study, we assessed the impact of the Israel–Iran conflict on the volume of ambulatory activity of a tertiary academic medical center located in central Israel.

## Methods

### Study design and settings

Sheba Medical Center is the largest hospital in Israel, with 1,944 inpatient beds, 2,000 physicians, and 3,321 nurses. This tertiary academic center includes seven institutions: a general hospital, rehabilitation center, psychiatric hospital, children’s hospital, women’s health center, cancer center, and Sheba Beyond Virtual Hospital. It performs over 6,000 outpatient visits daily across more than 150 specialties.

During the COVID-19 pandemic in 2020, Sheba rapidly integrated telehealth into routine outpatient care to maintain service continuity during lockdowns. This led to the establishment of a dedicated telehealth unit responsible for implementation, technical and administrative support, system integration, and clinical governance. Institutional guidelines were developed and disseminated to all clinical staff to ensure safe, standardized delivery of virtual care across both telephone- and video-based encounters. These protocols defined requirements for the clinical environment, patient identification, documentation standards, escalation pathways for urgent or deteriorating cases, and communication practices to support effective patient engagement. Institutional and national guidelines were implemented to standardize practice, and patient satisfaction has remained high, at approximately 90% annually [8].

Ambulatory activity is continuously monitored using the Sheba CCC (Care Control Center), a computerized real-time analytics system. It tracks key performance indicators including visit volume, no-show rates, telehealth versus in-person encounters, waiting times, encounter duration, and treatment delays, with data stratified by hospital and department.

### Israel–Iran conflict and its impact on hospital’s activity

Although the Israeli healthcare system had been operating under conflict conditions since October 2023, the impact on ambulatory activity in healthcare centers in central Israel was minimal. However, during the recent Israel–Iran conflict, multiple direct missile strikes occurred in central Israel, including three direct hits within a one-mile radius of Sheba Medical Center over the course of a single week.

A central component of maintaining ambulatory services during the conflict was the rapid expansion of telemedicine. A telehealth encounter was defined as a clinical interaction between a patient and a healthcare provider conducted remotely via telephone or video conferencing. This expansion was achieved through a coordinated operational strategy combining a telehealth-first workflow, proactive conversion of scheduled in-person visits to remote encounters, dedicated technical support, and centralized real-time monitoring of telemedicine activity. Many encounters were generated through same-day or day-before conversion of scheduled appointments in response to changing security conditions or patient inability to attend the medical center, enabling rapid scaling of remote care while preserving continuity of care.

Patients participated in telehealth encounters from home or another private setting, while clinicians conducted encounters from the clinic or, when feasible, from home. The choice between telephone- and video-based encounters was determined predominantly by clinical considerations and provider judgment. Video visits were used when visual assessment was required for clinical decision-making, such as dermatologic evaluation or assessment of range of motion, whereas telephone visits were used when visual information was not essential, offering a simpler and more accessible alternative for both patients and providers.

Telemedicine implementation followed a structured telehealth-first approach across ambulatory care. Clinical and administrative teams reviewed upcoming schedules to identify patients suitable for telehealth based on clinical and logistical considerations. Suitability for telehealth encounters was determined by the treating physicians, who reviewed the planned patient list for the following week and identified individuals appropriate for remote management. In many clinics, predefined criteria were used to support this selection process, and these criteria were reviewed and validated on a monthly basis to ensure ongoing clinical appropriateness. Priority for telemedicine conversion was given to selected clinical domains, including oncology, pediatrics, and obstetrics and gynecology. Oncology patients were prioritized due to the repeated and longitudinal nature of follow-up visits, while pediatrics and obstetrics and gynecology were prioritized due to the relatively young and often technologically adaptable patient populations, which facilitated effective remote engagement. Patients living far from the medical center or facing substantial travel burden were also preferentially considered.

Administrative staff proactively contacted patients before appointments, offering telehealth as the default option while allowing postponement when appropriate. SMS-based instructions and clear explanations improved acceptance and reduced uncertainty.

The digital infrastructure supporting this rapid scale-up was based on the routine integration of the DATOS platform for synchronous video-based telemedicine encounters and the Q-Flow system for queue management, scheduling orchestration, and patient flow coordination. These systems functioned as interconnected operational layers, enabling interoperability between appointment scheduling, clinician worklists, and real-time patient routing. The infrastructure supported secure patient authentication, standardized access to virtual encounters, and dynamic adjustment of schedules in response to fluctuating demand and security constraints. Together, these platforms enabled scalable, high-throughput delivery of both virtual and hybrid ambulatory care while maintaining continuity of operations during the crisis.

The Medical Center’s quality assurance teams conducted structured reviews of medical records of patients undergoing telemedicine visits. These audits assessed both encounter quality and the identification of safety-related events or risks to patient care. Risk governance was further supported by dedicated risk management meetings, in which cases with potential safety implications were reviewed promptly. These included situations requiring communication of significant pathology results or complex clinical decisions. When necessary, cases were reclassified as unsuitable for telemedicine and converted to in-person evaluations. These reviews also informed provider seniority decisions, with most encounters conducted by senior physicians rather than residents to ensure appropriate clinical oversight.

Organizational and behavioral strategies were implemented to support clinician engagement and adoption of telemedicine. Comparative analyses of telemedicine utilization were regularly presented to senior management and incorporated into annual departmental work plan approvals. In addition, an updated dashboard displaying telemedicine activity was made accessible to all managers, enabling transparency and ongoing performance feedback across clinical units. Clinicians were also enabled to conduct telemedicine encounters from home when operationally feasible, reducing travel burden and increasing flexibility in service delivery.

Implementation was supported by continuous performance monitoring and dedicated telehealth support teams. Daily hospital-level review of telemedicine utilization included visit volumes and proportional use across departments and sites. Comparative analyses identified high- and low-performing clinical areas, enabling targeted interventions, resource allocation, and dissemination of best practices. Dedicated support teams provided real-time technical assistance to clinicians, reducing barriers and facilitating integration of telemedicine into routine workflows.

### Data collection and definitions

We calculated the number of ambulatory visits across ten hospital divisions—surgery, internal medicine, oncology, obstetrics and gynecology, rehabilitation, psychiatry, pediatrics, cardiovascular, neurology and neurosurgery, and anesthesia and pain—during the seven weekdays of the Israel–Iran war. Weekends were excluded to enable appropriate comparison with routine operational periods. Visit counts were then compared with those recorded on the corresponding calendar dates (16–25 June) in the four years preceding the current war (2021–2024). All encounters were classified as either in-person or telemedicine-based.

Institutional Review Board approval was waived for this study. The study’s findings are reported in accordance with the Strengthening the Reporting of Observational Studies in Epidemiology (STROBE) guidelines [9].

### Statistical analysis

We summarized annual visit volumes and telemedicine utilization using descriptive statistics, reporting counts and proportions for each year from 2021 to 2025. The primary outcome was the annual proportion of visits conducted via telemedicine (telemedicine visits divided by total outpatient visits).

To evaluate the effect of the onset of the Israel–Iran war on telemedicine use, we employed an interrupted time series design with segmented linear regression. Years 2021–2024 were defined as the pre-war period and 2025 as the war period. For each year, the telemedicine proportion was modeled as a function of a continuous time index and an indicator for the war year. This approach enabled estimation of the underlying pre-war temporal trend and the immediate level change in telemedicine use associated with the onset of war in 2025. Ordinary least squares regression was used to fit the model, and 95% confidence intervals (95% CI) were calculated using a t-distribution. Effect estimates for the war indicator are reported as absolute percentage-point changes in the telemedicine proportion with corresponding 95% CIs. A *p*-value of <0.05 was considered statistically significant. All analyses were performed using the Statistical Package for the Social Sciences (SPSS), version 23.0 (IBM Corp., Armonk, NY, USA).

## Results

During seven working days between June 13 and June 24, 2025, a total of 53,158 ambulatory visits were conducted at Sheba Medical Center, of which 9,478 (17.8%) were delivered via telemedicine. Of these telehealth encounters, 85% were conducted by telephone and 15% via video, compared with 71 and 29%, respectively, during the pre-war period. Half of the patients (26,581) were male, and 47.8% (25,430) were between 25 and 64 years old. The demographic characteristics of the patients treated during this period, as well as the distribution of visits across the clinical departments, are presented in Table 1. A substantially higher proportion of in-person visits were made by male patients compared with telemedicine visits (55.6% vs 24.2%, *p* < 0.001). The age distribution differed significantly between in-person and telemedicine visits (*p* < 0.001). In-person encounters were disproportionately used by middle-aged and older adults (25–64 and ≥65 years), whereas telemedicine visits were relatively more common among younger patients. Additionally, most in-person visits were from patients living in the central district (86.2%), compared with less than half of telemedicine visits (44.6%; *p* < 0.001), indicating greater telemedicine use among patients residing outside the central district.

**Table 1:** Demographic characteristics of 53,158 ambulatory visits conducted during the study period (June 13 and June 24, 2025) as well as their distribution across the clinical departments

	Visits conducted in person (n = 43,680)	Telemedicine (n = 9,478)
**Male gender, n (%)**	24,284 (55.6%)	2,297 (24.2%)
Age, n (%)*		
Children (0–17 years)	4,524 (10.4%)	1021 (10.8%)
Young adults (18–24 years)	3,439 (7.9%)	285 (3%)
Adults (25–64 years)	22,868 (52.4%)	2562 (27%)
Elderly (≥65 years)	16,854 (38.6%)	1548 (16.3%)
**Patients living in the central district, n (%)**	37,673 (86.2%)	4229 (44.6%)
**Clinical department, n (%)**		
Oncology	8,178 (18.7%)	2,424 (25.6%)
Internal Medicine	7,958 (18.2%)	1,673 (17.7%)
Surgery	7,992 (18.3%)	464 (4.9%)
Rehabilitation	4,043 (9.3%)	75 (0.8%)
Pediatrics	2,747 (6.3%)	1,849 (19.5%)
Cardiovascular	3,507 (8%)	558 (5.9%)
Obstetrics and Gynecology	3,284 (7.5%)	444 (4.7%)
Psychiatry	2,055 (4.7%)	1,052 (11.1%)
Neurology and Neurosurgery	1,990 (4.6%)	473 (5%)
Anesthesia and Pain	1,059 (2.4%)	382 (4%)
Other	866 (2%)	84 (0.9%)

^*^Age data were missing for 57 patients (0.1%)

**Fig. 1:**
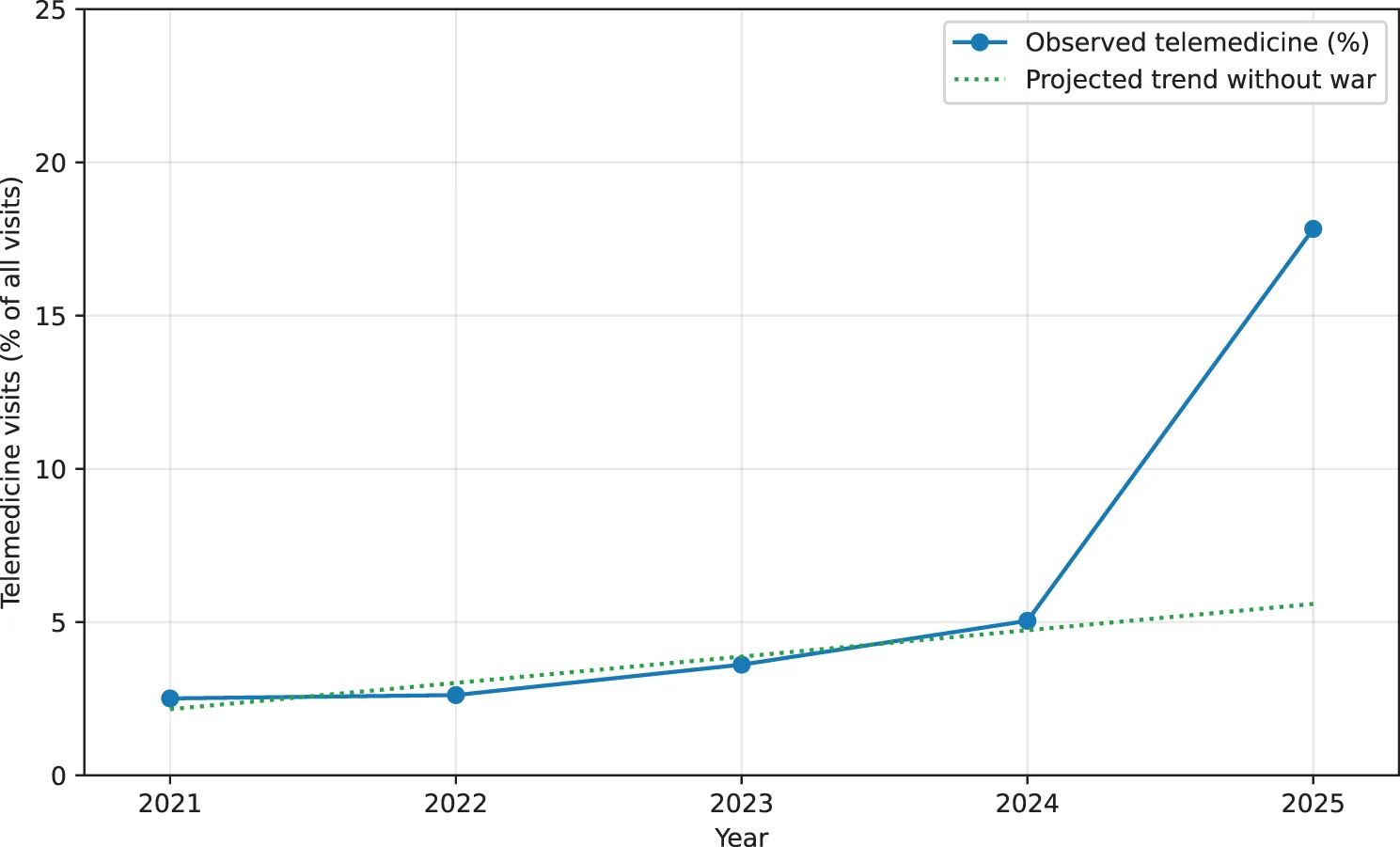
Annual proportion of outpatient visits conducted via telemedicine during weekdays between June 13 and 24 from 2021 to 2025

From 2021 through 2025, there were 319,313 total outpatient visits during weekdays between June 13 and 24, of which 18,583 (5.8%) were conducted via telemedicine. During the pre-war period (2021–2024), telemedicine use increased gradually. Telemedicine visits rose from 1,841 of 73,402 total visits in 2021 (2.5%) to 3,350 of 66,469 visits in 2024 (5.0%), reflecting a slow but steady increase in the proportion of remotely conducted visits. In contrast, in 2025 telemedicine utilization increased sharply, reaching 17.8% of all visits. In the interrupted time series model, the estimated pre-war temporal trend in telemedicine use was an increase of approximately 0.86 percentage points per year. After accounting for this underlying trend, the Israel–Iran conflict was associated with a large and abrupt increase in telemedicine utilization. The estimated level change in the telemedicine proportion during the war was 12.2 percentage points (95% CI 9.0 to 15.5 percentage points). This indicates that, beyond the gradual increases observed from 2021 to 2024, the proportion of visits conducted via telemedicine in 2025 was roughly 12 percentage points higher than would have been expected if the pre-war trend had continued uninterrupted (Fig. 1).

**Fig. 2:**
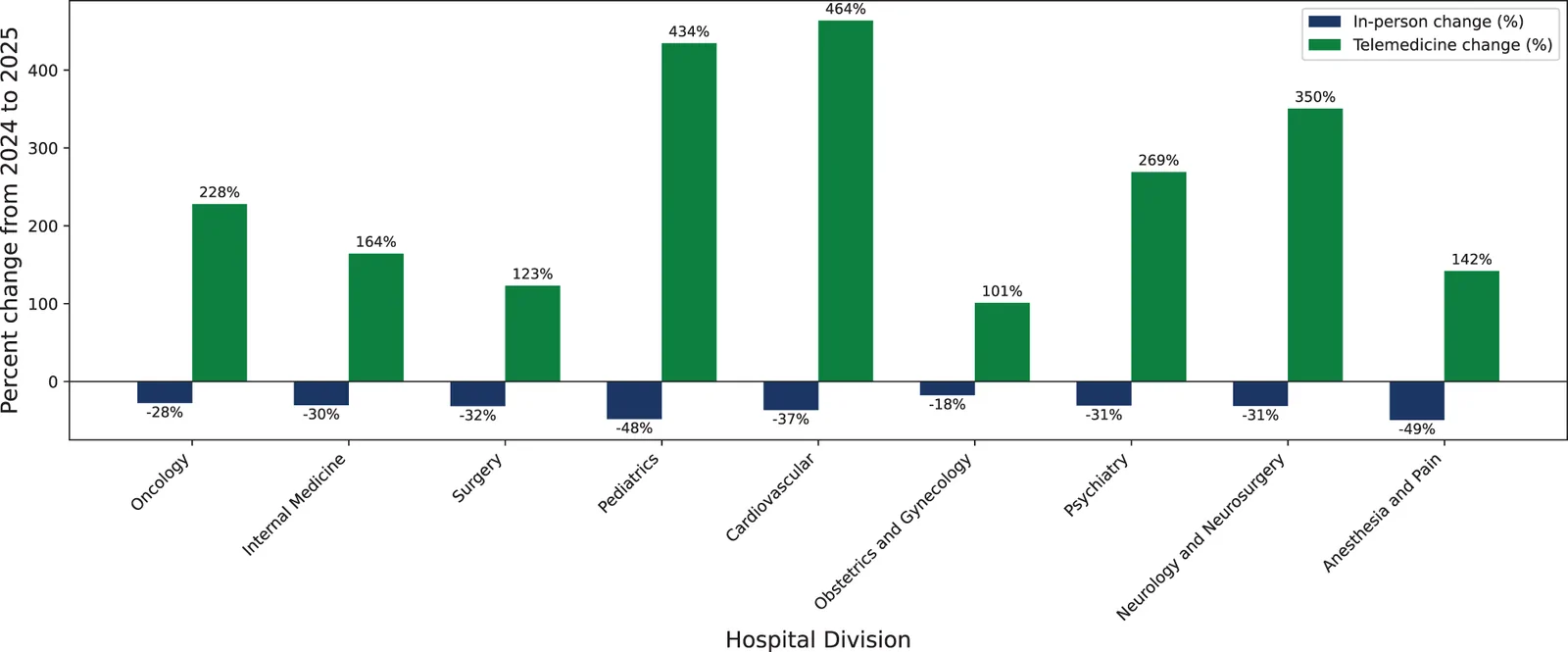
Percent change in in‑person and telemedicine visits by hospital division from 2024 to 2025

When comparing the study period with the same timeframe in 2024, the total number of outpatient visits decreased by 20.0% (from 66,469 to 53,158). Telemedicine visits increased by 182.9% (from 3,350 to 9,478), while in-person visits decreased by 30.8% (from 63,119 to 43,680). In In other words, the number of in-person visits decreased by approximately 20,000, while telemedicine visits increased by approximately 6,000, offsetting approximately 30% of the decline in in-person visits. The distribution of outpatient visits by clinical department is summarized in Table 2. In the pediatric hospital, 40.2% of appointments were conducted via telemedicine, compared with 6.1% in 2024 (*p* < 0.001). In the psychiatric wing, 33.9% of visits were telemedicine-based, versus 8.7% in 2024 (*p* < 0.001). As shown in Fig. 2, all hospital divisions experienced substantial decreases in in-person visits alongside corresponding increases in telemedicine visits from 2024 to 2025. The largest increases in telemedicine encounters were observed in the cardiovascular (463.6%), pediatric (434.4%), and neurology and neurosurgery (350.5%) divisions, while more modest increases occurred in anesthesia (141.8%), surgery (123.1%), and obstetrics and gynecology (100.9%).

**Table 2:** Proportion of telemedicine visits by clinical department from June 13–24, 2024, and June 13–24, 2025

	**2024** (*n* = 66,469)		**2025** (*n* = 53,158)		*p***-value**
	In person (*n* = 63,119)	Telemedicine (*n* = 3,350)	In person (*n* = 43,680)	Telemedicine (*n* = 9,478)	
Oncology, n (%)	11,307 (93.9%)	739 (6.1%)	8,178 (77.1%)	2,424 (22.9%)	<0.001
Internal Medicine, n (%)	11,409 (94.7%)	633 (5.3%)	7,958 (82.6%)	1,673 (17.4%)	
Surgery, n (%)	11,696 (98.3%)	208 (1.7%)	7,992 (94.5%)	464 (5.5%)	
Rehabilitation, n (%)	5,839 (100.0%)	0 (0.0%)	4,043 (98.2%)	75 (1.8%)	
Pediatrics, n (%)	5,293 (93.9%)	346 (6.1%)	2,747 (59.8%)	1,849 (40.2%)	
Cardiovascular, n (%)	5,528 (98.2%)	99 (1.8%)	3,507 (86.3%)	558 (13.7%)	
Obstetrics and Gynecology, n (%)	3,986 (94.7%)	221 (5.3%)	3,284 (88.1%)	444 (11.9%)	
Psychiatry, n (%)	2,977 (91.3%)	285 (8.7%)	2,055 (66.1%)	1,052 (33.9%)	
Neurology and Neurosurgery, n (%)	2,900 (96.5%)	105 (3.5%)	1,990 (80.8%)	473 (19.2%)	
Anesthesia and Pain, n (%)	2,092 (93.0%)	158 (7%)	1,059 (73.5%)	382 (26.5%)	
Other, n (%)	92 (14.2%)	556 (85.8%)	866 (91.2%)	84 (8.8%)

## Discussion

During the current conflict, which affected civilian areas nationwide, our hospital maintained routine outpatient services at approximately 80% of average daily activity. This was likely facilitated by the marked 183% increase in telemedicine appointments.

During crises, hospitals primarily prioritize maintaining emergency services over ambulatory care, particularly in the early phase, resulting in limited comparative data on outpatient service preservation. Sberro-Cohen and Ellen reported increased telemedicine utilization during the October 7, 2023, war in Israel without detailing ambulatory volume changes [10], while Bernstine et al., also from Israel, focused on emergency department activity, hospitalizations, and outcomes without addressing outpatient services [11]. Hadary et al. described only modest reductions in outpatient activity- a 5% decrease in outpatient clinic visits- during the Second Lebanon War at Ziv Medical Center [1]. In 74 Ukrainian hospitals across 12 oblasts, a 10% reduction in daily outpatient services (from 10,853 to 9,769 daily visits) was reported during the Russia–Ukraine War [12]. The larger reduction observed in our study (20%) may be explained by the relatively short and highly acute study period, which likely did not allow sufficient time for adaptive behavioral or organizational stabilization of outpatient activity. In addition, multiple direct missile strikes in central Israel likely contributed to the higher observed decline in ambulatory volume, exceeding that described in more prolonged or geographically limited conflicts.

Wars and other disasters can significantly disrupt healthcare services. Medically vulnerable populations—such as the elderly, uninsured, and individuals with chronic conditions—often suffer disproportionately poor health outcomes following such events and may face prolonged delays in regaining access to care [4, 13, 14]. Community hospitals located within military conflict zones are tasked with treating both civilian and military casualties while simultaneously maintaining routine medical and surgical services for the broader community [1]. Many patients seek care and medications for pre-existing conditions rather than acute issues, yet medical personnel often lack the necessary supplies or specialized training to adequately address these needs [15, 16].

With the increasing frequency of severe weather events and military conflicts worldwide, it is critical for the healthcare community to develop strategies that can accommodate the volume and diversity of patient needs during such crises, while maintaining ambulatory and elective services. Effective approaches to maintaining ambulatory services and surge capacity during wars and disasters focus on four key domains: staff, supplies (“stuff”), space, and systems. Proven strategies include the development of flexible and scalable disaster response plans, enhanced coordination and communication across departments and agencies, and the implementation of structured incident management systems to optimize resource utilization and ensure adaptability to various types of crises [17–19]. Our findings further suggest that successful telemedicine deployment during crises depends not only on the availability of technology but also on its integration into routine clinical practice before emergencies occur. Healthcare resilience requires adaptive capacity during crises, which is heavily dependent on organizational preparedness and operational infrastructure established during normal periods. In our experience, maintaining ambulatory services during wartime required considerable administrative agility, including the same-day conversion of scheduled in-person visits to telemedicine encounters, extension of staffing hours, rapid clinician enablement for remote work, and continuous operational monitoring through centralized digital dashboards. These observations highlight practical steps that may help other healthcare institutions strengthen preparedness, including investment in telemedicine infrastructure, workforce training, flexible scheduling systems, and organizational processes that allow rapid transitions between in-person and virtual models of care.

Telemedicine plays a critical role in managing short-term surge capacity for ambulatory and routine care during wars and disasters by enabling the remote delivery of medical services. This approach preserves access to care when in-person visits are disrupted or unsafe. As a force multiplier, telemedicine allows clinicians to extend their reach, provide both specialty and primary care consultations, and maintain continuity of care for patients with chronic and acute conditions, even in high-conflict or infrastructure-compromised environments [10, 20]. The American College of Chest Physicians recommends telemedicine as an essential adjunct to disaster response, emphasizing its capacity to support both ambulatory and critical care, facilitate remote patient monitoring, and alleviate pressure on overwhelmed facilities by managing stable patients outside the hospital setting [2]. Empirical evidence from recent conflicts and disasters show that telemedicine utilization rises significantly during crises, while maintaining high patient satisfaction and enhancing system adaptability across primary care, mental health, and specialty services [10, 21]. Furthermore, prior familiarity with telemedicine is associated with reduced emergency department (ED) visits and inpatient admissions for ambulatory care–sensitive conditions, underscoring its preventive role and contribution to preserving healthcare system capacity [4].

During the current conflict, 40% of appointments in the children’s hospital were conducted via telemedicine. This level of telemedicine use was likely supported by the demographic characteristics of the pediatric population and their families, who tend to be more familiar and comfortable with digital health technologies. In the psychiatric department—where physical examinations are often unnecessary—34% of appointments were performed via telemedicine. Telemedicine has proven effective in managing mental health conditions such as post-traumatic stress disorder, anxiety, and depression, which tend to be highly prevalent during times of crisis [22]. Telemedicine also serves as a means of delivering psychological support to the broader public, potentially mitigating the development of conditions like post-traumatic stress disorder [23]. In pediatric primary care, a recent large-scale study found that while telemedicine visits were associated with modestly higher rates of subsequent in-person and ED visits, they did not result in higher hospitalization rates. Telemedicine appears to be a valuable tool for healthcare delivery in the pediatric population, though it is not a complete substitute for in-person care [24].

The increased use of telemedicine during wartime was documented by Israeli health maintenance organizations during the October 7, 2023, conflict. Consistent with our findings, the most pronounced rise was observed in mental health consultations, while family medicine, pediatrics, and gynecology also showed notable increases in telemedicine utilization.[10]

This study has several limitations. First, it was conducted at a single tertiary medical center in central Israel, which may limit the generalizability of the findings to other settings, particularly those with different infrastructure, resources, or security conditions. Second, the analysis focused on ambulatory visit volumes and modes of delivery but did not assess clinical outcomes, patient satisfaction, or quality of care. Third, due to the retrospective nature of the study and the urgency of data collection during an ongoing conflict, certain contextual factors—such as patients’ reasons for choosing telehealth over in-person visits—could not be systematically captured. Finally, it should be noted that Sheba Medical Center has been operating under conflict conditions since October 2023. Although the impact on the hospital’s ambulatory activity appears limited, it cannot be ignored.

## Conclusion

In this study, we demonstrated that continuity of ambulatory care can be maintained during armed conflict through strategic use of telemedicine, protected infrastructure, and proactive patient engagement. Telehealth can sustain ambulatory care during conflict, when in-person services are limited by security threats or infrastructure constraints. A structured “telehealth-first” approach, supported by digital tools, enables near-continuous care. Early integration into pre-conflict preparedness, with coordination between administrative and clinical teams, is critical. Continuous monitoring of utilization and performance supports adoption and quality improvement. Data-driven strategies, including artificial intelligence, can further optimize patient selection and telehealth engagement.

## Data Availability

The datasets used and/or analysed during the current study are available from the corresponding author on reasonable request.

## Abbreviations

95% CI: 95% Confidence intervals
ED: Emergency department
